# The Effect of Biochar and Its Interaction with the Earthworm *Pontoscolex corethrurus* on Soil Microbial Community Structure in Tropical Soils

**DOI:** 10.1371/journal.pone.0124891

**Published:** 2015-04-21

**Authors:** Jorge Paz-Ferreiro, Chenfei Liang, Shenglei Fu, Ana Mendez, Gabriel Gasco

**Affiliations:** 1 School of Civil, Environmental and Chemical Engineering, RMIT University, Melbourne, Australia; 2 Key Laboratory of Vegetation Restoration and Management of Degraded Ecosystems, South China Botanical Garden, Chinese Academy of Sciences, Guangzhou, China; 3 University of Chinese Academy of Sciences, Beijing, China; 4 Departamento de Ingeniería Geológica y Minera, ETSI Minas, Universidad Politécnica de Madrid, Madrid, Spain; 5 Departamento de Producción Agraria, ETSI Agrónomos, Universidad Politécnica de Madrid, Madrid, Spain; Tennessee State University, UNITED STATES

## Abstract

Biochar effects on soil microbial abundance and community structure are keys for understanding the biogeochemical cycling of nutrients and organic matter turnover, but are poorly understood, in particular in tropical areas. We conducted a greenhouse experiment in which we added biochars produced from four different feedstocks [sewage sludge (B1), deinking sewage sludge (B2), Miscanthus (B3) and pine wood (B4)] at a rate of 3% (w/w) to two tropical soils (an Acrisol and a Ferralsol) planted with proso millet (*Panicum milliaceum* L.). The interactive effect of the addition of earthworms was also addressed. For this purpose we utilized soil samples from pots with or without the earthworm *Pontoscolex corethrurus*, which is a ubiquitous earthworm in tropical soils. Phospholipid fatty acid (PLFA) measurements showed that biochar type, soil type and the presence of earthworms significantly affected soil microbial community size and structure. In general, biochar addition affected fungal but not bacterial populations. Overall, biochars rich in ash (B1 and B2) resulted in a marked increase in the fungi to bacteria ratio, while this ratio was unaltered after addition of biochars with a high fixed carbon content (B3 and B4). Our study remarked the contrasting effect that both, biochar prepared from different materials and macrofauna, can have on soil microbial community. Such changes might end up with ecosystem-level effects.

## Introduction

Seeking for new technologies to improve agricultural systems and contributing to sustainable resource management has led to a number of creative ideas, including the pyrolysis of biomass residues (biochar) and their addition to the soil system. Much of the modern interest in biochar research comes from the investigation of Amazonian Dark Earths or “Terra Preta”, a type of anthropogenic soil with an elevated content of pyrogenic organic matter. As a consequence of the beneficial role of biochar in Amazonian Dark Earths, there was an increasing interest on biochar research in the last years, mainly fuelled by its significance with respect to the abatement of carbon emissions [1], increase in agricultural yields [2] and improvement in the utilisation of wastes [3]. In addition, several studies have shown how a general improvement in soil physical, chemical and biological activities can be expected from the use of different biochars [4, 5].

Tropical areas present a high potential to increase yield following biochar addition [2]. In general, the use of tropical soils for agriculture is constrained by low organic matter contents, CEC and nutrient contents, in particular for Acrisols and Ferralsols [6], and this can be improved with biochar addition. For example, Peng et al. [7] found an increment in pH and CEC of an Ultisol after addition of rice straw-derived biochar and Yin et al. [8] demonstrated that the application of biochar to soil may be an appropriate management practice for increasing soil C storage in Ferrasol. Research in biochar has been mostly focused on its use as a soil conditioner altering soil physical and chemical properties with less attention given to its effect on soil biological properties. In the last years, some pioneer studies have demonstrated that biochar could increase soil enzymatic activity [4] but, in the particular case of tropical soils, this increase is very specific and depends on the soil type, biochar and enzyme activity studied [5].

There are some studies on the effect of biochar on soil microbial activity and community structure. However, and in particular when referred to community structure, these studies are limited to soils in the temperate region [9, 10, 11, 12, 13] or in Terra Preta soils [14], but not in tropical soils amended with modern-made biochars. Factors studied by these authors include biochar concentration [10, 13, 15] and pyrolysis temperature [11, 12]. In a limited number of studies the authors have considered more than one feedstock [11, 12]. However, these experiments usually do not study a large number of biochars with a range of properties.

On the other hand, earthworms alter soil physical, chemical and biological properties, through grazing, burrowing and casting and dispersal. Earthworms alter soil structure and fertility, through their direct and indirect contribution to the creation of organic structures (biopores, biogenic aggregates) which alter microbial mineralization of organic matter and nutrient cycling [16] and can also change soil enzymatic activity [5]. This could lead to drastic shifts in the microbial communities within the drilosphere or the bulk soil [17]. Moreover, in the last years there has been some attention devoted to the interaction between earthworms and biochar. Earthworms can not only ingest soil but also biochar [18], which could alter its distribution in the soil profile or biochar intrinsic properties. Earthworm-biochar interactions have been addressed in the context of soil protein turnover [19], plant resource allocation [20], enzyme activity [5] or greenhouse gas emissions [21], but not with respect to microbial community structure. It has also been suggested that earthworms and, in particular, grinding and mixing activity of the circumtropical species *Pontoscolex corethrurus*, could have favoured the genesis of Amazonian Dark Earths [22].

Taking into consideration the abovementioned issues, the aim of this experiment was to study the effect of biochar addition and its interaction with earthworm activity on soil microbial community and structure in two tropical soils, an Acrisol and a Ferralsol. We hypothesized that 1) Biochar would have a positive effect in microbial biomass and activity and would shift community composition, but the effect will be dependent on biochar properties and soil type 2) Earthworms and biochar could interact in the shifts observed in microorganism communities.

## Materials and Methods

### Soils and biochar

The soils were sampled at Heshan Hilly Land Interdisciplinary Experimental Station, Chinese Academy of Sciences in Guangdong Province, China, located at 22°41′N and 112°54′E in March of 2012. The sampling procedure, climate of the area and soil types (an Acrisol and a Ferralsol) as well as the four biochars in this study have been thoroughly described in a previous study [5].

B1 and B2 were produced from sewage sludge and deinking sludge, respectively, and produced by slow pyrolysis at a temperature of 600°C. B3 was produced from Miscanthus at a pyrolysis temperature of 450°C and B4 was produced from wood using gasification at a temperature of 800°C. Table 1 shows the main properties of the biochars used in this experiment. The four biochars used in this experiment differed greatly from each other. B3 and B4 were characterized by a very high carbon content, but they differed in terms of pH, EC and trace element content. B1 and B2 proximate analyses revealed an elevated proportion of ash contents. Both biochars had lower surface area and carbon contents than B3 and B4. The highest trace metal contents were in B1 as a consequence of the parent material. The C/N ratio followed the order B3>B2>B4>B1.

**Table 1:** General properties of the biochars analysed.

	Acrisol	Ferralsol	B1	B2	B3	B4
pH	3.35	5.0	8.50	10.31	6.12	12.15
EC (μS cm^-1^)			1460	236	420	7160
C (%)	3.35	1.53	11.3	14.0	65.4	74.2
N(%)	0.253	0.140	0.787	0.199	0.604	1.313
C/N	13	11	14.3	70.3	108.2	56.5
Surface area (m^2^ g^-1^)			37	36	89	169
Volatile matter (%)			16.70	32.97	49.67	17.00
Fixed carbon (%)			4.77	2.22	31.58	53.18
Ash (%)			78.53	64.81	18.75	29.82
Cu (mg kg^-1^)			740	136	4.8	595
Ni (mg kg^-1^)			134	22	13	298
Cd (mg kg^-1^)			9.76	0.2	n.a.	1.8
Zn (mg kg^-1^)			3922	54	18	1361
Sand (%)	52	54				
Silt (%)	17	21				
Clay (%)	31	25				
Texture	Sandy-clay-loam					

The experiment was conducted at a greenhouse in the South China Botanical Garden, Guangzhou. Pots (diameter 10 cm and 15 cm height) were filled with 400 g of soil (sieved to 5 mm). The experiment was replicated four times. Biochars were added to the soil at a rate of 3% (w/w), selected for its potential to increase plant productivity [5]. Moreover, this application rate was similar to those used in other studies [10, 14]. Afterwards, soils were planted with eight seeds of proso millet (*Panicum milliaceum* L.). Two weeks after planting, three adult individuals of *Pontoscolex corethrurus* (Annelida: Oligochaeta: Glossoscolecidae) were introduced at each mesocosm. All the earthworms were sampled from the same site as the Ferrasol. Soils were maintained at 60% soil water holding capacity, which was checked through regular weighing of the pots. Mesocosms were arranged in a completely randomized design inside the greenhouse. Soil sampling took place 3 months after the beginning of the experiment. After sampling about 100 g of soil, samples were freeze-dried for PLFA analysis and another part was stored in a fridge at 4°C for microbial biomass and soil respiration.

### Methods

Microbial biomass carbon was determined by the fumigation-extraction method using 10 g of soil [23]. The difference in C contents between the fumigated and unfumigated extracts was converted to microbial biomass C using a conversion factor (*Kc*) of 0.45.

Microbial respiration was determined by static incubation. The CO_2_ evolved in 10 days from 25 g soil samples incubated at 60% water holding capacity (WHC) and at 25°C was collected in 10 ml of a 1 *M* NaOH solution, which was then titrated with HCl.

Microbial community structure was assessed by analyzing the composition of extractable ester-linked PLFAs, using the method was described by Bossio and Scow [24]. Concentrations of individual PLFAs were calculated based on 19:0 internal standard concentrations. The indicator PLFAs were used for classification of microbial community types.

The fatty acids i15:0, a15:0, i16:0, i17:0, a17:0, cy17:0, 18:1ω7, 18:1ω5 and cy19:0 were chosen to represent bacterial PLFAs. Of these, gram-positive bacteria were indicated by i15:0, a15:0, i16:0, i17:0 and a17:0, while gram-negative bacterial PLFA included cy17:0, 18:1ω7, 18:1ω5 and cy19:0. In addition, 18:2ω6 was used as an indicator of fungal biomass [25]. Actinomycetes were represented by 10Me16:0, 10Me17:0 and 10Me 18:0 [25] while arbuscular mycorrhizal fungi (AMF) were indicated by 16:1ω5c [26].

### Statistical analyses

All statistical analyses were performed using SPSS 15.0. A three-way ANOVA was carried out using the different soil parameters measured as variables and type of biochar (derived from sewage sludge, deinking sewage sludge, Miscanthus or pine wood), soil type (Acrisol or Ferralsol) and earthworm (presence or absence) as factors. A post-hoc analysis using the Tukey test was conducted for the factor “biochar”. Unless otherwise stated, the differences were significant at P <0.05 level.

## Results

**Fig 1:**
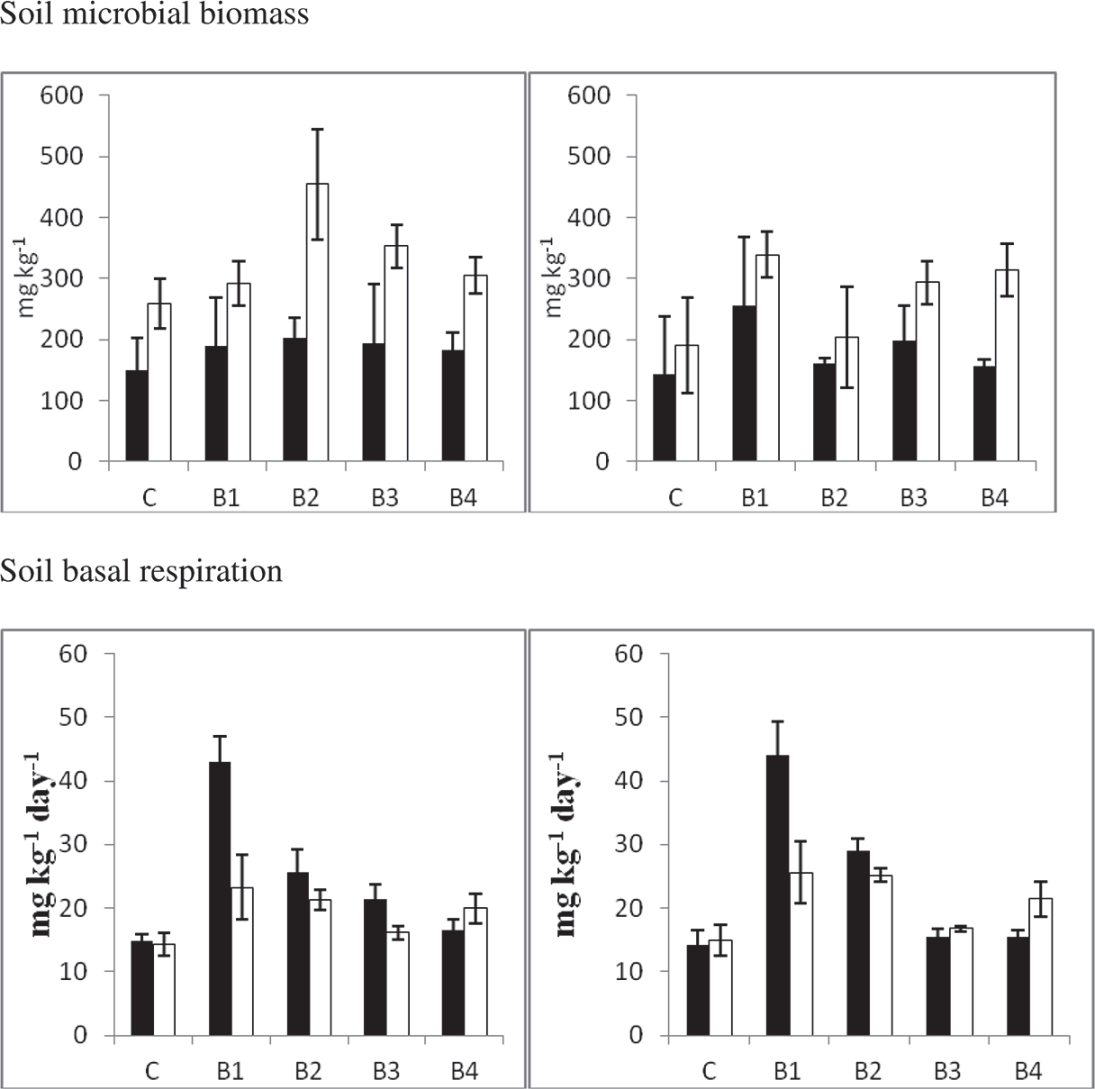
Soil microbial biomass C and soil basal respiration. Left and right graph represent treatments with and without earthworms, respectively. Filled bars represent the Acrisol, while white bars represent the Ferralsol.

Fig 1 shows the results for soil microbial biomass C. This property was affected by soil type (F = 72.3, P<0.001, Table 2), with greater values in the Ferralsol (300±87 mg kg^-1^) compared to the Acrisol (183±66 mg kg^-1^). The effect of the amendment was statistically significant (F = 4.6, P<0.010) and the values of microbial biomass were also higher in soils amended with B1, B2 and B3 than in the control soil (see Table 3). In addition, values of microbial biomass were higher in the mesocosms with earthworms (283±104 mg kg^-1^) than in the treatments without earthworms (183±66 mg kg^-1^) (F = 5.7, P<0.05). However, it should be noted here that the presence of earthworms had an effect on soil biomass in the Ferralsol but not in the Acrisol. In fact the F value of the soil x earthworm interaction was 5.5 (P<0.05). There was also a significant interaction between biochars and earthworms (F = 5.6, P <0.001) explained by earthworms having a stronger effect on soils amended with biochar B2 with respect to other biochars.

**Table 2:** F values for the different properties and effects considered.

	Biochar (B)	Soil (S)	Earthworm (E)	BxS	BxE	SxE	BxSxE
Soil microbial biomass	**4.6 (0.003)**	**72.3 (<0.001)**	**5.7 (0.020)**	0.9 (0.437)	**5.6 (<0.001)**	**5.5 (0.023)**	2.1 (0.088)
Soil respiration	**126.4 (<0.001)**	**43.7 (<0.001)**	0.7 (0.392)	**41.9 (<0.001)**	**2.9 (0.031)**	**4.1 (0.046)**	0.7 (0.564)
Bacterial biomass	1.2 (0.315)	0.3 (0.592)	**10.0 (0.002)**	**15.6 (<0.001)**	1.7 (0.158)	<0.1 (0.902)	1.7 (0.171)
Fungal biomass	**3.0 (0.027)**	**74.3 (<0.001)**	3.8 (0.057)	**10.3 (<0.001)**	2.4 (0.058)	0.6 (0.461)	1.9 (0.115)
Actinomycetes	2.5 (0.055)	**9.2 (0.004)**	**4.5 (0.038)**	**17.2 (<0.001)**	2.5 (0.054)	0.3 (0.565)	1.1 (0.377)
AM fungi	**4.2 (0.005)**	**161.8 (<0.001)**	**6.6 (0.013)**	**13.0 (<0.001)**	1.5 (0.026)	<0.1 (0.915)	2.3 (0.070)
Bacterial to fungal ratios	**7.9 (<0.001)**	**173.0 (<0.001)**	0.032 (0.858)	1.5 (0.205)	1.2 (0.309)	0.2 (0.661)	1.8 (0.134)
Gram+ to gram- bacteria	**7.7 (<0.001)**	**18.3 (<0.001)**	<0.1 (0.952)	**12.0 (<0.001)**	1.4 (0.235)	**6.9 (0.011)**	1.7 (0.170)

P values are shown in brackets. Values in bold indicate that the differences are statistically significant (3-way ANOVA, P<0.05).

**Table 3:** Average values and standard deviation for the properties analized in this study.

	Control	B1	B2	B3	B4
Soil microbial biomass	186±78 a	269±87 b	255±133 b	259±87 b	239±78 ab
Soil respiration	14.54±1.82 a	33.95±10.79 d	25.27±3.49 c	17.46±2.74 b	18.33±3.11 b
Bacterial biomass	7.70±1.36 a	7.59±3.23 a	8.42±1.23 a	8.36±0.85 a	7.96±1.89 a
Fungal biomass	1.23±0.59 a	1.44±0.48 ab	1.62±0.57 b	1.47±0.65 ab	1.32±0.56 ab
Actinomycetes	1.86±0.33 a	1.78±0.72 a	1.78±0.26 a	2.09±0.39 a	1.96±0.57 a
AM fungi	0.34±0.13 a	0.41±0.12 ab	0.44±0.12 b	0.41±0.17 ab	0.44±0.22 b
Fungi to bacteria ratio	0.15±0.05 a	0.20±0.05 b	0.20±0.06 b	0.17±0.06 ab	0.16±0.04 a
Gram+ to gram- bacteria	2.57±0.11 ab	2.78±0.45 c	2.73±0.32 bc	2.46±0.07 a	2.71±0.18 bc

Different letters in the same row are indicating statistical significant differences (3-way ANOVA, P<0.05). Each mean value for control, B1, B2, B3 and B4 is the average of 16 values (two soils x presence or absence of earthworms x 4 replicates).

Soil respiration increased following biochar addition (F = 126.4, P<0.001, Fig 1) and was higher in the Acrisol than in the Ferralsol (F = 43.7, P<0.001). There was a strongly significant soil x biochar interaction (F = 41.9, P<0.001). Thus, B1 and B2 increased soil respiration in the Acrisol, while B1, B2 and B4 increased respiration in the Ferralsol. In addition, this upsurge in soil respiration after B1 addition was more prominent in the Acrisol than in the Ferralsol. Earthworm addition increased or maintained respiration in the Acrisol, but diminished this same property in the Ferralsol (F = 4.1, P<0.05 for earthworm x soil interaction, data not shown). There was also a slightly significant biochar x earthworm interaction (F = 2.9, P<0.05).

**Fig 2:**
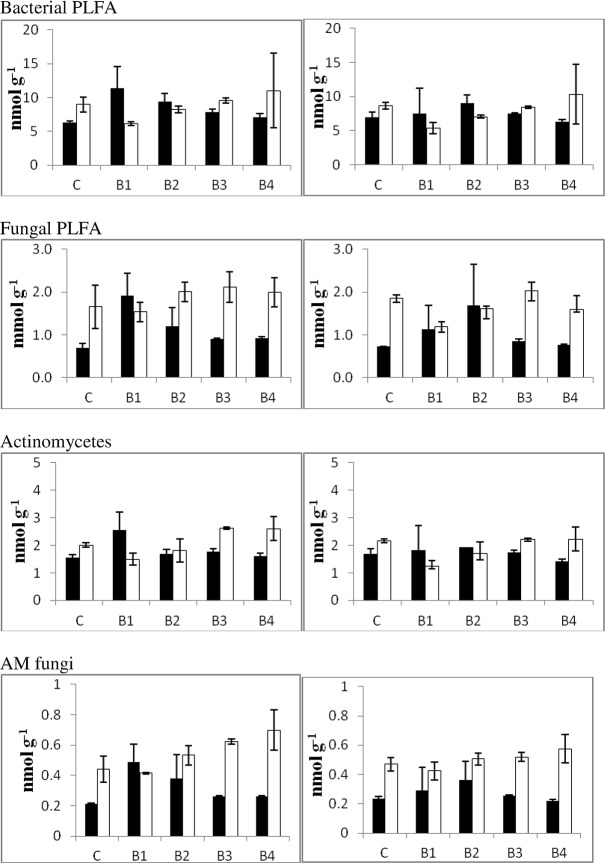
Bacterial, fungal, AM fungi and actinomicetes PLFA. Left and right graph represent treatments with and without earthworms, respectively. Filled bars represent the Acrisol, while white bars represent the Ferralsol.

Fig 2 shows PLFA results. The amount of bacterial PLFA was influenced by the presence of earthworms (F = 15.6, P<0.001) with a value of 8.49±2.00 and 7.52±1.64 nmol g^-1^ dry soil in samples with and without earthworms, respectively. There was a strongly significant soil x biochar interaction (F = 15.6, P<0.001). Thus, biochar amendment resulted in slight increases of soil bacterial PLFA when B1 and B2 were added to the Acrisol. In contrast with these results, B1 decreased bacterial PLFA following its addition to the Ferralsol.

The amount of fungal PLFA was affected by soil type (F = 74.3, P<0.001) and by the presence of biochar (F = 3.0, P<0.05). Fungal PLFA value was 1.76±0.37 in the Ferralsol compared to 1.45±0.5464 nmol g^-1^ dry soil in the Acrisol. In addition, biochar effects on this activity depended on soil type, as there was a significant soil x biochar interaction (F = 10.3, P<0.001). Thus, fungal PLFA was higher after amendment with B1 and B2 in the Acrisol but biochar had not a significant effect in the Ferralsol.

Actinomycetes averaged 1.97±0.51 nmol g^-1^ dry soil in samples with earthworm versus 1.81±0.45 nmol g^-1^ dry soil in samples without earthworms. This difference was statistically significant (F = 4.5, P<0.05). Actinomycete concentration in the Ferralsol (2.01±0.51 nmol g^-1^ dry soil) was higher than in the Acrisol (1.78±0.43 nmol g^-1^ dry soil, F = 9.2, P<0.01). There was a statistically significant biochar x soil interaction (F = 17.2, P<0.001). In particular, B1 reduced actinomycete population in the Ferralsol, while no effect was detected for other amendments in either the Ferralsol or the Acrisol.

Arbuscular mycorrhizal (AM) fungi were influenced by biochar addition (F = 4.2, P<0.01), they increased in the presence of earthworms (F = 6.6, P<0.05) and exhibited higher values in the Ferralsol compared to the Acrisol (F = 161.8, P<0.001). In addition, there was a significant biochar x soil interaction (F = 13.0, P<0.001). While B1 and B2 increased AM fungi in the Acrisol, this effect was only attained by B4 in the Ferralsol.

**Fig 3:**
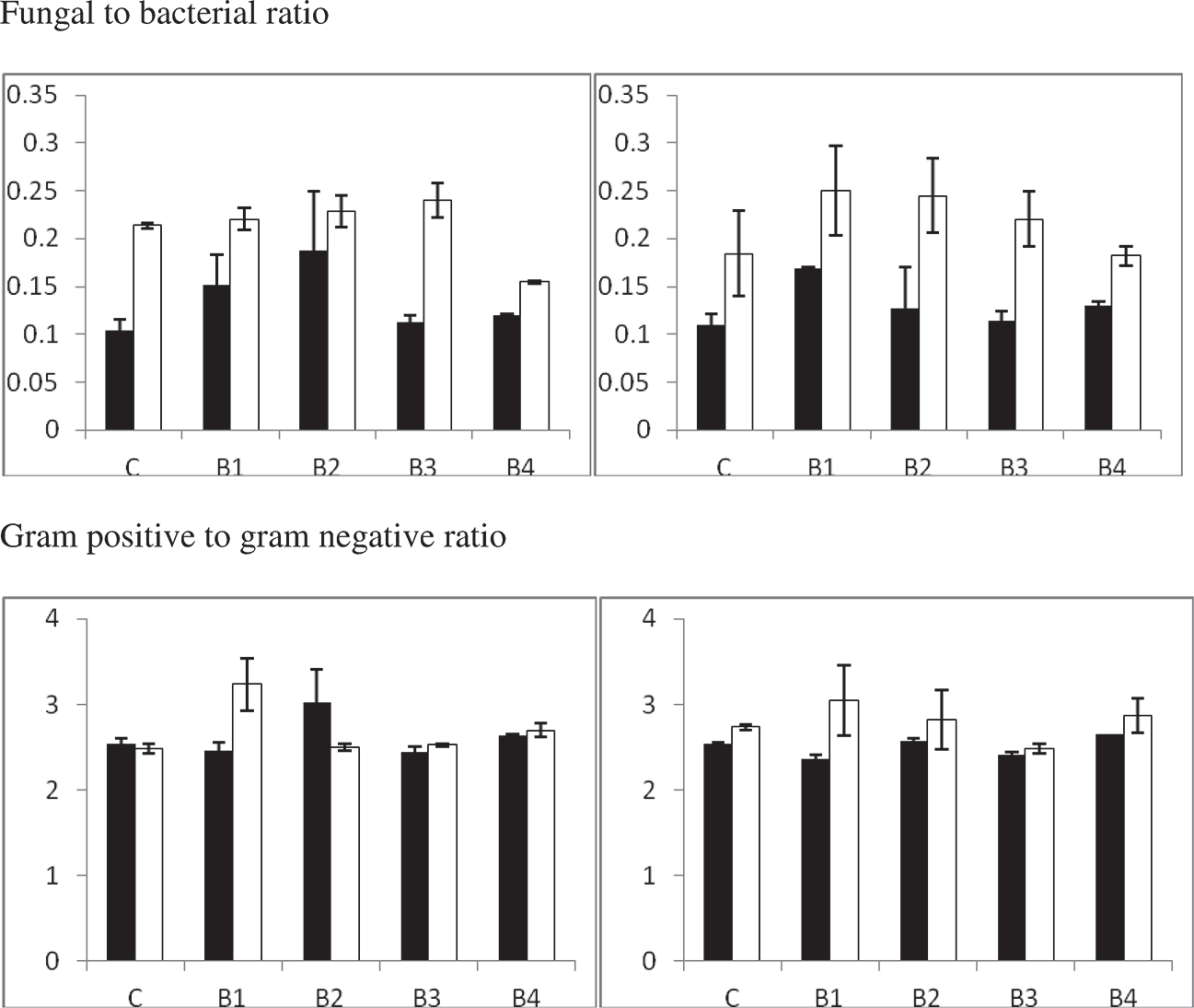
Fungal to bacterial ratio and gram positive to gram negative ratio. Left and right graph represents treatments with and without earthworms, respectively. Filled bars represent the Acrisol, while white bars represent the Ferralsol.

Fig 3 shows Fungal to bacterial ratio and gram positive to gram negative ratio. The ratio of fungal to bacterial biomass was affected by biochar (F = 7.9, P<0.001) with values following the following order: B1 = B2>B3>B4 = C. Values of fungal to bacterial ratio averaged 0.22±0.03 for the Ferralsol and 0.13±0.04 for the Acrisol (F = 172.5 and P<0.001 for soil effect).

The ratio of gram-positive to gram-negative bacteria was altered by biochar addition (F = 7.7, P<0.001) and by soil type (F = 18.3, P<0.001). There was a statistically significant biochar x soil interaction (F = 12.0, P<0.001) and also a significant earthworm x soil interaction (F = 6.9, P<0.05). Thus, gram-positive to gram-negative bacteria ratio increased with B2 addition in the Acrisol but not in the Ferralsol. The ratio of gram-positive to gram-negative bacteria averaged a value of 2.74±0.31 in the Ferralsol and 2.56±0.23 in the Acrisol.

## Discussion

We tested the effect of biochar addition in two soil types differing in soil organic matter content, pH and fertility (Table 1). Overall, our results confirm previous studies carried out in temperate latitudes that have found that biochar addition to soil results in an stimulation of soil microbial communities, which can be measured by the fumigation-extraction method or by PLFA or result in higher soil enzyme activity [3, 10]. However, on occasions a negative effect of some biochars on soil microbial properties has been reported [14]. Soil basal respiration, a measure of the activity of soil microbial communities also increased following biochar addition to the soil. The mechanisms behind the positive effect of biochar in soil microbial activity are diverse and include improved pH after biochar addition to the soil, physical properties of biochar which result in an increase in surface area that can be colonized by soil microorganisms and possibly an increase in soil water holding capacity. In this sense it seems reasonable to conclude that in our soils, both chemical (pH) and physical (surface area) factors were important in relation to the total size of the soil microbial community. Few differences were found in microbial biomass C after the addition of biochars that altered the most soil pH, B1 and B2 (see [5]) or after addition of biochars with a high surface area, B3 and B4. This result contradicts the expectations of increased pH and/or surface positive effects on microbial abundance [27]. However, stronger trends were found after exploring the community composition in detail.

The biochars used in our study had an effect on AM fungi and fungal biomass, but their effect on bacteria or actinomycetes were soil-specific (see Table 3). Our results contrast with those of Gómez et al. [10] who found that bacteria, fungi and actinomycetes showed a significant increase after biochar addition. AM fungi are known to be obligate rhizospheric symbionts found in the vast majority of crop plants. In general, AM biomass was found to be higher in soils were pH increases were most noticeable. This dependence of AM fungi on pH has also been reported previously in a variety of ecosystems [28, 29]. The limited literature available in relation with biochar effects on soil microbial communities does not allow to discern which communities are mainly affected by biochar additions. It seems that the type of biochar chemical structure can offer an advantage to fungal or bacterial organisms, depending on the intrinsic properties of the biochar, a result in accordance to Steinbeiss et al. [9]. While Steinbeiss et al. [9] found that the influence of the initial soil type on the microbial community after soil-biochar interaction was negligible; we found an important effect of the initial soil type.

A shift to a more fungal-dominated biomass after biochar addition was observed in our study. Moreover, this alteration was driven mostly by an increase in the soil fungal community, rather than by changes in the bacterial community (see Table 3). Biochar addition has been hypothesized to result in a preferential advantage for fungi due to the importance of fungi in the degradation of lignin [30], although the opposite trend has also been stated [27] as bacteria are favoured over fungi after pH increases associated to biochar addition in acidic soils. However, recent articles seemed to disproof this hypothesis. As an example both Gómez et al. [10] and Ameloot et al. [12] reported decreases in fungal to bacterial ratios after biochar amendment, while Prayogo et al. [13] found no changes in this ratio. The fungal to bacterial ratios of the soil biomass were much more affected by B1 and B2 rather than B3 and B4 addition. Increases in fungal biomass have been reported in soils amended with biomass ashes [31] and were associated with an increase in carbon availability, a mechanism that is likely to have occurred in our ash-rich materials B1 and B2. Increased fungal to bacterial ratios are hypothesized to be indicative of more sustainable agricultural systems, with important benefits including a more efficient crop nutrient uptake mediated by mycorrhizal fungi [32]. It is also necessary to stress that this and other research on the topic are short-term incubation studies and their results could not be totally extrapolated to the field. In fact, tillage, an event that can disrupt soil structure, affects primarily fungi, as their mycelia can be broken up, instead of bacteria. Thus, it could be possible that, under field conditions, fungal communities do not experience the strong effect reported in our work under laboratory conditions.

Gram positive to gram negative ratios increased or remained constant after biochar addition in our samples. These results contrast to [10, 13] who found that gram negative bacteria were the community predominantly stimulated by biochar addition. Our study indicates the importance not only of biochar type with respect to the preferred energy source of microbial communities but also of soil type.

We found that mesocosms with earthworms exhibited a greater microbial biomass, which is unsurprising as endogeic earthworms, such as *Pontoscolex corethrurus*, modify microbial biomass through digestion, stimulation and dispersion in casts [33, 34]. Previous studies have shown that earthworms can exert a strong influence on soil microbial community composition as a consequence of enhanced nutrient mineralization, which is a consequence of earthworm activity [35, 36]. As an example, we have found that AM fungi and bacterial biomass increased in the mesocosms containing earthworms. Actinomycetes also increased with the presence of earthworms, an expected result as they have been reported to be selectively stimulated in the earthworm gut [17].

With the notable exceptions of soil microbial biomass and soil respiration we did not observe a statistically significant interaction between biochar addition and the presence of earthworms (see Table 2). Our soils are representative of the tropical area and, in particular, Southern China. Thus, given the lack of interaction between the factors biochar rate and earthworms for most of the properties analyzed, we presume that these two factors are to some extent, but not totally, independent.

Biochar mediated microbial community compositional changes deserve further research, as these alterations can have essential implications for carbon sequestration and biogeochemical cycling. This is due to distinctive groups of microbial organisms being related to different biogeochemical processes. As an example fungal to bacterial ratios have been linked to the resistance and resilience of soil microbial communities to disturbances such as drought [37], while Gram-positive bacteria and Gram-negative bacteria have different functions in soils, differing for example in their use of carbon sources. Thus, while gram positive bacteria use predominantly complex carbon sources derived from soil organic matter, gram negative bacteria tend to use more plant litter [38].

Our study, in combination with previous work [5] stresses that the addition of different biochars could be used to improve soil productivity without causing adverse effects on the size of the microbial community. More research will be needed to understand the implications of biochar altering the structure of the microbial community.

## Conclusions

Our study implies that both biochar and earthworm can alter soil microbial community and structure in tropical soils. In general, amendment with biochar had a greater effect than earthworm addition on soil microbial community. However, the earthworm effect and sometimes a biochar-earthworm significant interaction means that most experiments done with biochar, which remove earthworms before laboratory incubation, could be misinterpreting biochar mediated changes in microbial communities. The effect of biochars with poor organic contents (B1 and B2) was very distinctive compared with those of biochars rich in fixed carbon (B3 and B4), in particular in terms of their impacts on the fungal and microbial communities. Overall, a higher fungal to bacterial ratio was detected for biochars B1 and B2. Our results hint that biochar addition to soil can alter microbial functioning and soil productivity, with broad implications for soil biogeochemical processes, but those results are dependent on the soil-biochar interaction, which is highly specific.
